# A comparative analysis of semen quality traits and sperm kinematic parameters in relation to fertility prediction in Murrah buffaloes

**DOI:** 10.5455/javar.2025.l938

**Published:** 2025-08-18

**Authors:** Syahruddin Said, Athhar Manabi Diansyah, Johana Dian Rahayu, Tulus Maulana, Muhammad Gunawan, Ekayanti Mulyawati Kaiin

**Affiliations:** 1 Research Center for Applied Zoology, National Research and Innovation Agency, Cibinong, Indonesia; 2 Faculty of Animal Science, Hasanuddin University, Makassar, Indonesia

**Keywords:** Reproductive assessment, buffalo reproduction, CASA analysis immunofluorescence, reproductive efficiency

## Abstract

**Objective:**

The study aims to identify the most reliable fertility indicators, contributing to the development of more precise and effective evaluation protocols for Murrah buffalo breeding programs.

**Materials and Methods:**

This study analyzed 120 cryopreserved semen samples from four Murrah buffaloes. Each sample underwent quality evaluation, and sperm kinematics were analyzed using computer-assisted semen analysis. Analysis of variance was used to compare sperm quality between different bull IDs. Pearson's correlation was used to measure the correlation between each parameter.

**Result:**

The statistical analysis indicated that the motility of sperm from bull M001 was markedly reduced (*p* < 0.05) relative to bull M003. Likewise, the viability of sperm in M001 was significantly diminished (*p* < 0.05) compared to other bulls. The mitochondrial membrane potential in sperm from M004 was significantly elevated (*p* < 0.05) over that of M003. Furthermore, membrane integrity in M004 sperm showed a significant increase (*p* < 0.05) compared to M001. Kinematic measurements for buffalo M003 exhibited significantly greater (*p* < 0.05) values than those of M002. A strong inverse correlation (*r *= – 0.972) was identified between sperm quality and kinematic variables through statistical analysis, particularly involving linearity and DNA fragmentation, with significance confirmed at *p* < 0.05.

**Conclusion:**

The sperm quality of Murrah buffalo differs among the four buffaloes examined, particularly regarding sperm motility, viability, and energy potential. Among kinematic parameters, only velocity average path showed significant variation. The study also revealed a relationship between sperm with more linear movement and better DNA integrity.

## Introduction

The Murrah buffalo is widely valued for its exceptional milk production and adaptability to various environments. However, maintaining high and consistent fertility rates remains a major challenge, as fluctuations in fertility can significantly impact the productivity and sustainability of breeding programs [1]. Breeding soundness evaluation (BSE) is commonly used to assess the reproductive potential of bulls, including those of the Murrah breed [2].

Semen quality is evaluated by BSE using conventional markers of sperm health and viability, including morphology, motility, and concentration [3]. However, BSE has limitations, particularly in predicting actual fertility outcomes, as it focuses primarily on static sperm characteristics and does not account for the dynamic aspects of sperm movement, which are essential for successful fertilization [4, 5].

A key limitation of BSE is its focus on static sperm characteristics, overlooking critical dynamic traits such as progressive speed and navigation ability, which are essential for successful fertilization. For example, sperm with high motility and normal morphology in laboratory conditions may not perform effectively in natural reproductive settings [6, 7]. This disconnect can lead to discrepancies between BSE results and actual fertility outcomes in the field.

Environmental and physiological variations in Murrah buffalo can influence the outcomes of BSE, making the results inconsistent and difficult to interpret. To address these challenges, a more comprehensive evaluation method is needed—one that integrates traditional semen quality assessments with sperm kinematic analysis to provide more reliable fertility predictions [8]. The limitations of BSE underscore the need for advanced reproductive assessment methods, such as sperm kinematic analysis. This approach offers more details about sperm movement characteristics, including speed, linearity (LIN), and progressive motility (PM), which are essential for successful fertilization [9, 10].

Integrating conventional BSE methods with sperm kinematic analysis offers a more holistic evaluation of reproductive potential in Murrah buffalo. Strong correlations between sperm kinematics and fertility have been reported in previous studies, suggesting that combining these approaches can overcome BSE limitations [11]. However, limited research has focused on this integration in Murrah buffalo. The study aims to identify the most reliable fertility indicators, contributing to the development of more precise and effective evaluation protocols for Murrah buffalo breeding programs [12].

This study carries major implications for the field of animal reproduction. By addressing the limitations of traditional BSE through the incorporation of sperm kinematic analysis, it aims to establish a more comprehensive and precise fertility assessment method, not only for Murrah buffaloes but also for other livestock species. The ultimate goal is to enhance the efficiency and sustainability of breeding programs, which are essential for ensuring the productivity and resilience of future livestock populations.

## Materials and Methods

### Ethical approval

Cryopreserved semen samples were collected from a total of four Murrah bulls aged between 9 and 10 years, represented by six batches each containing five straws, amounting to 120 samples. These bulls had an average body weight ranging from 700 to 900 kg. Bull selection was determined by their health conditions and documented fertility performance according to records from the Artificial Insemination Center (AIC). Selected bulls were classified as superior based on their BSE outcomes. The National Research and Innovation Agency's Animal Ethics Commission approved the use of animal models and experimental procedures in this study, with certificate number 050/KE.02/SK/03/2023.  Following the guidelines laid down by the Indonesian National Standard (SNI 4869-2: 2021), the semen was then frozen, according to the methods outlined by Herbowo et al. [13].

### Frozen semen quality

Before being placed into microtubes for further examination, cryopreserved semen samples from every bull were thawed in a water bath kept at 37°C for 30 sec. The following parameters for sperm quality were investigated in this study: acrosome integrity, DNA fragmentation, protamine insufficiency, anomalies, viability, progressive motility (PM), and sperm motility [14].

### Viability and abnormality

Arif et al. [15] provided the modified approach that was used to evaluate sperm viability and abnormalities. To determine viability, a glass slide was stained with a mixture of 50 µl of thawed semen and 100 µl of eosin-nigrosin solution. The slides were viewed under a light microscope at a magnification of 400x after thorough homogenization and drying on a heating plate. Each sample was analyzed over 10 microscopic areas, including roughly 200 spermatozoa. Viable sperm remained unstained, but non-viable sperm heads exhibited red staining, indicating membrane integrity failure. Morphological abnormalities were assessed using the Diff-Quick Staining Kit (Aurora Scientific, Indonesia) in accordance with the methodology outlined by Tuset et al. [16]. Sperm abnormalities were classified as head, neck, midpiece, and tail problems [17].

### Mitochondrial membrane potential (MMP)

The MMP was evaluated by applying TMRE staining according to the BD Pharmingen guidelines. Cells (≤ 1 × 10^6^/ml) were stained with 200 nM TMRE for 15–30 min at 37°C, washed, resuspended, and analyzed by flow cytometry (BD AccuriTM C6 Plus) at 575/26 or 582/15 nM. Data acquisition (50,000 events) was stored for analysis [18].

### Membrane integrity

Evaluation of plasma membrane integrity was performed employing the hypo-osmotic swelling (HOS) assay. Sperm exhibiting intact plasma membranes were identified by the presence of curled or swollen tails, whereas straight tails signified membrane damage or sperm death. This was determined through microscopic examination of 200 spermatozoa per sample at 400x magnification [19].

### Acrosome integrity

The sperm's intact acrosome was assessed using FITC-PNA and propidium iodide (PI) fluorescence staining. Semen smears were air-dried, fixed in 96% ethanol, and incubated with 30 μl PNA (100 μg/ml) at 37°C for 30 min. Afterward, 5 µl P.I. (1 µg/µl) was applied for 5 min, followed by PBS rinsing. Acrosome status was evaluated under a fluorescence microscope (380–420 nm) by analyzing 200 sperm cells per treatment. Green fluorescence indicated an intact acrosome, while reddish staining signified a damaged acrosome [20].

### DNA fragmentation

The evaluation of DNA fragmentation in spermatozoa was conducted using the Sperm-Bos-Halomax^®^ kit (Halotech DNA, SL, Spain) in accordance with the manufacturer's instructions. A sperm concentration of 15 million per ml was generated and mixed with the solvent, while the agarose gel was liquefied at 95°C for 5 min and then equilibrated at 37°C. 25 μl of sperm suspension was combined with 50 μl of agarose solution; thereafter, 2 μl of this mixture was pipetted onto coated slides, covered with coverslips, and incubated at 4°C for 5 min. 

Subsequent to coverslip removal, the slides were subjected to lysis treatment, rinsed with distilled water, dehydrated with ethanol, and stained with propidium iodide (P.I.). Following washing with PBS and air drying, two hundred spermatozoa were analyzed using a fluorescence microscope (Imager Z2, Carl Zeiss) at 63x magnification. Sperm cells were categorized according to halo formation patterns: A prominent halo signified intact DNA, whereas the absence of a halo, lysed cells lacking halos, or the presence of a small, dense halo indicated DNA fragmentation.

### Protamine deficiency

The chromomycin A3 (CMA3) staining method was applied to evaluate protamine deficiency [21]. The samples underwent two washes with phosphate-buffered saline (PBS). Subsequently subjected to centrifugation and fixation in Carnoy's solution for 8 min, the samples were then spread onto APES-coated slides and allowed to air dry. Before CMA3 staining, all processes were carried out at a temperature of 4°C. The slides were left to incubate for 30 min with 100 µl of a CMA3 solution containing 0.25 mg/ml, made in McIlvaine buffer (pH 7.0) with 10 mM MgCl_2_ added. Antifade solution (Fluoprep, BioMerieux, France) was applied after rinsing in McIlvaine buffer and air drying. Fluorescence microscopy at 460–470 nm was used for observation, where sperm showing protamine deficiency fluoresced bright yellow, whereas sperm with normal protamine exhibited dark or dull yellow fluorescence.

### Kinematic sperm

A warming stage kept at 38°C and 400x magnification was used to evaluate sperm motility and kinematics using computer-assisted semen analysis (CASA) software, namely the Sperm-Vision Program (Minitüb, Tiefenbach, Germany), in conjunction with Carl Zeiss Microimaging GmbH equipment (Göttingen, Germany). Here are some of the parameters that were measured: total motility (TM), PM, distance straight line (DSL), average path distance (DAP), curvilinear velocity (VCL), velocity straight line (VSL), average path velocity (VAP), LIN, straightness (STR), and wobble (WOB) [22]. Roughly 1,000 spermatozoa per sample were used to determine the mean values for each parameter [23].

### Data analysis

The kinematic and sperm quality data were shown as means with standard deviations. The requirements for performing ANOVA and Pearson correlation tests were met before the research began by ensuring that the data were normalized using the Shapiro–Wilk test and that the variances were homogeneous using Levene's test. Tukey's post hoc test was used to detect statistically significant changes between pairings after one-way ANOVA was utilized to examine mean sperm quality and kinematic features across different bull IDs. To evaluate the relationships between the measured variables, we used the Pearson correlation. Version 25 of IBM SPSS Statistics for Windows (IBM Corp., Chicago, IL, USA) was used for all statistical analyses.

## Results

### Sperm quality and kinematics of Murrah buffaloes

**Figure 1.**
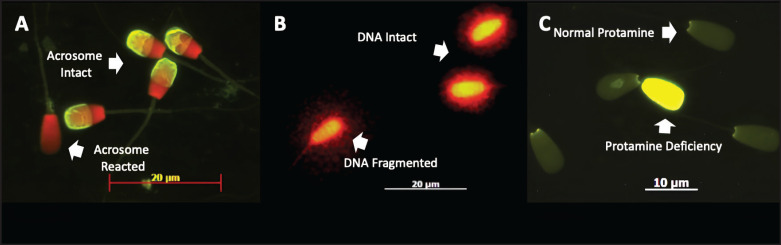
Magnification 630x, microscope fluorescence, Imager Z7, Carl Zeiss, Germany. A. Acrosome integrity. The acrosome is intact (green fluorescence in the acrosome); the acrosome reacts (red fluorescence). B. DNA integrity. DNA intact (small or null halo); DNA fragmented (large halo). C. Protamine status. Normal protamine (dark/dull green fluorescence); protamine deficiency (yellow fluorescence).

Table 1 indicates substantial differences (*p *< 0.05) among the bulls for motility, viability, MMP, and membrane integrity. Specifically, bull M001 showed the lowest sperm viability (65.53%) and membrane integrity (63.82%), whereas bulls M003 and M004 exhibited significantly higher values ranging from 71.77% to 71.88% and 67.84% to 67.95%, respectively. The highest MMP was observed in bull M004 (99.03%), while bull M003 presented the lowest value (87.67%). There were no significant changes (*p* > 0.05) in traits like progressive motility, sperm abnormalities, acrosome integrity, DNA fragmentation, and protamine insufficiency, as shown in Figure 1.

The data presented in Table 2 showed that most sperm kinematic parameters of Murrah buffalo bulls did not differ significantly (*p* > 0.05), except for VAP, which varied significantly (*p* < 0.05) among the bulls. Bulls M003 and M004 exhibited the highest VAP values, measuring 78.24 and 75.87 µm/sec, respectively, whereas bull M002 recorded the lowest value at 66.16 µm/sec.

### The correlation between sperm quality and kinematics in Murrah buffaloes

**Figure 2.**
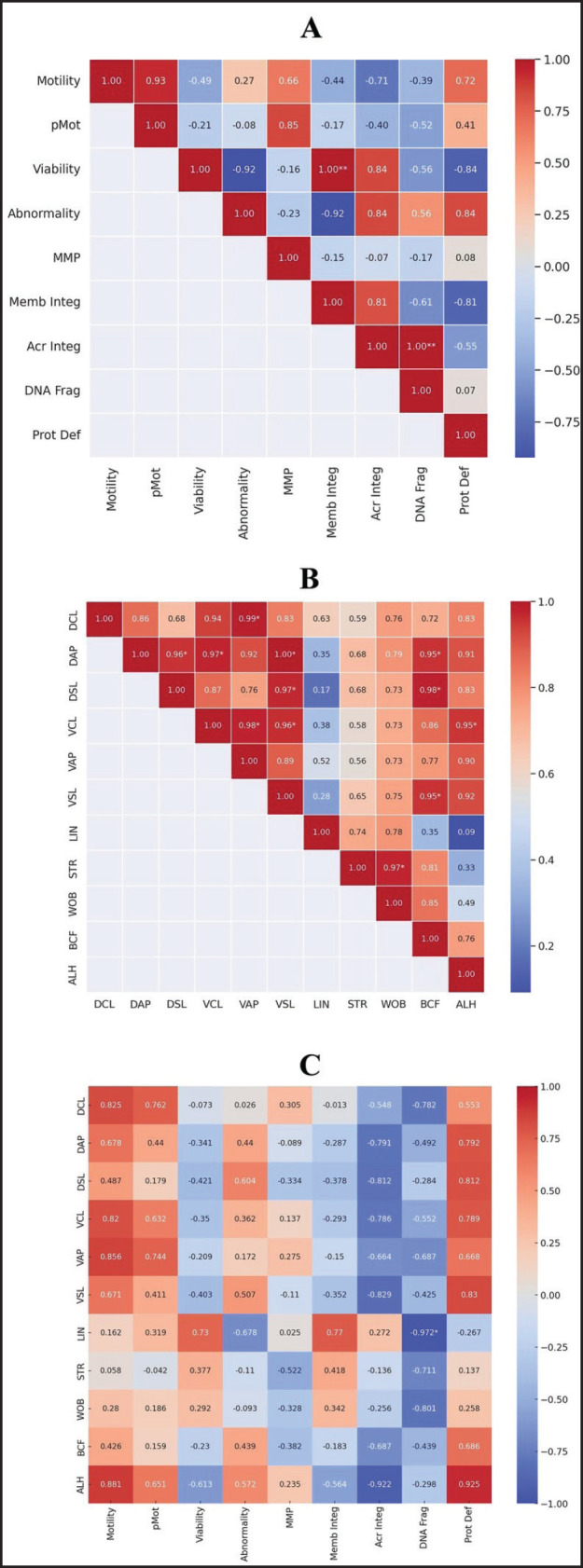
Heatmap visualization of Pearson’s correlation of sperm quality on Murrah buffaloes. (A) The correlation between sperm quality. (B) The correlation between sperm kinematics. (C) The correlation between sperm quality and kinematics. The scale is based on color from red (positive) to blue (negative); *Significant correlation (*p* < 0.05); **Significant correlation (*p* < 0.01); very strong (0.76–0.99); strong (0.51–0.75); fair (0.26–0.50); weak (0.00–0.25).

This study analyzes the correlation between sperm quality and kinematics in Murrah buffaloes. The correlation from each parameter on Murrah buffaloes is presented in Figure 2.

**Table 1.** The sperm quality of Murrah buffalo bulls.

Parameter	ID Bull			
	M001	M002	M003	M004
Motility (%)	62.43 ± 3.84^a^	63.47 ± 3.63^ab^	66.47 ± 2.17^b^	65.26 ± 1.45^ab^
Progressive motility (%)	49.17 ± 15.49^ns^	49.59 ± 14.63^ ns^	55.65 ± 2.87^ ns^	57.14 ± 1.47^ ns^
Viability (%)	65.53 ± 2.8^a^	69.01 ± 2.01^b^	71.77 ± 4.06^b^	71.88 ± 2.09^b^
Abnormality (%)	7.10 ± 1.10^ ns^	6.66 ± 0.77^ ns^	6.56 ± 1.16^ ns^	6.20 ± 0.73^ ns^
Mitochondrial membrane Potential (%)	95.17 ± 6.99^ab^	93.70 ± 7.69^ab^	87.67 ± 16.29^a^	99.03 ± 0.12^b^
Membrane integrity (%)	63.82 ± 3.12^a^	65.84 ± 2.21^ab^	67.84 ± 1.92^b^	67.95 ± 1.22^b^
Acrosome integrity (%)	94.04 ± 1.43^ ns^	95.37 ± 0.68^ ns^	95.16 ± 1.02^ ns^	95.32 ± 0.96^ ns^
DNA fragmentation (%)	6.67 ± 0.83^ ns^	7.84 ± 1.96^ ns^	5.91 ± 1.93^ ns^	5.26 ± 0.74^ ns^
Protamine Deficiency (%)	5.96 ± 1.43^ ns^	4.63 ± 0.68^ ns^	4.84 ± 1.02^ ns^	4.69 ± 0.96^ ns^

Different superscripts in the same row indicate a significant difference (*p* < 0.05). Data are presented in the mean ± SD.

Figure 2A illustrates several significant positive correlations (*p* < 0.05), including very strong relationships (*r* = 0.76–0.99) between variables such as sperm viability and membrane integrity, highlighting a robust association among these quality indicators. Additionally, other parameters demonstrated highly significant positive correlations (*p* < 0.01), supporting the notion that enhanced sperm quality corresponds with improved structural and functional integrity of the sperm membrane. Conversely, some correlations were weak (*r* = 0.00–0.25) and statistically non-significant (*p* > 0.05), for example, between sperm concentration and various quality measures.

A Pearson's correlation study of sperm kinematic characteristics in Murrah buffaloes is shown in Figure 2B. Numerous noteworthy positive correlations were found in the study (*p* < 0.05), with particularly robust correlations (*r* = 0.76–0.99) between VCL and VAP. In addition, there were significant associations (*p* < 0.01) found between VCL and both VSL and amplitude of lateral head displacement (ALH), as well as VAP and VSL. These results indicate close interrelations among kinematic parameters, suggesting that improvements in one may positively influence others, thereby enhancing overall sperm motility. However, weak correlations (*r* = 0.00–0.25) without statistical significance (*p* > 0.05) were observed between beat cross frequency (BCF) and other parameters, indicating less strong associations in these cases.

Figure 2C presents Pearson’s correlation between sperm quality and kinematic parameters, underscoring complex interactions critical for assessing sperm performance in Murrah buffaloes. Strong positive correlations (*r* = 0.76–0.99) were observed among distance curvilinear (DCL), VCL, VAP, and ALH. In contrast, weaker correlations existed between ALH, membrane integrity, acrosome integrity, and DNA fragmentation. These kinematic factors showed strong associations with quality metrics such as motility, viability, DNA fragmentation, membrane integrity, and abnormalities, suggesting that higher sperm quality relates closely to more efficient sperm movement.

**Table 2.** The sperm kinematics of Murrah buffaloes.

Parameter	ID Bull			
	M001	M002	M003	M004
DCL (μm)	44.64± 4.97^ns^	39.44 ± 5.69^ ns^	47.18± 6.94^ ns^	46.81± 6.84^ ns^
DAP (μm)	32.11 ± 3.37^ns^	28.01 ± 3.85^ ns^	33.85 ± 3.80^ ns^	30.93 ± 3.99^ ns^
DSL (μm)	24.60 ± 4.67^ ns^	20.93 ± 4.59^ ns^	25.74 ± 5.66^ ns^	22.15 ± 5.16^ ns^
VCL (μm/sec)	101.85 ± 9.98^ ns^	91.82 ± 10.36^ ns^	109.05 ± 14.41^ ns^	102.37 ± 12.15^ ns^
VAP (μm/sec)	73.39 ± 6.57^ab^	66.16 ± 5.50^a^	78.24 ± 9.66^b^	75.87 ± 5.52^ab^
VSL (μm/sec)	55.96 ± 8.74^ ns^	48.95 ± 8.52^ ns^	59.44 ± 11.95^ ns^	53.51 ± 10.84^ ns^
LIN (%)	54.50 ± 6.56^ ns^	53.00 ± 4.55^ ns^	53.50 ± 5.32^ ns^	54.75 ± 5.32^ ns^
STR (%)	75.50 ± 8.81^ ns^	74.00 ± 6.53^ ns^	74.75 ± 6.13^ ns^	74.75 ± 7.50^ ns^
WOB (%)	71.75 ± 2.50^ ns^	71.00 ± 0.82^ ns^	71.50 ± 3.11^ ns^	71.50 ± 2.89^ ns^
BCF (Hz)	29.97 ± 5.23^ ns^	25.94 ± 6.11^ ns^	30.19 ± 4.31^ ns^	27.63 ± 6.09^ ns^
ALH (mm)	4.45 ± 1.11^ ns^	4.34 ± 0.80^ ns^	4.67 ± 0.45^ ns^	4.48 ± 1.03^ ns^

ALH = amplitude of lateral head; BCF: beat cross frequency; DAP = distance average path; DCL = distance curve linear; DSL = distance straight line; VAP = velocity average path; VCL = velocity curvilinear; VSL = velocity straight line; STR = straightness; LIN = linearity; WOB = wobble. Different superscripts in the same row indicate a significant difference (*p* < 0.05). Data are presented in the mean ± SD.

Significant positive correlations between VCL, VAP, DCL, and ALH with motility (*r* = 0.82, 0.856, 0.825, and 0.881, respectively) suggest that the velocity, curvilinear path length, and lateral head movement amplitude play crucial roles in supporting active sperm motility. Most kinematic parameters were negatively correlated with DNA fragmentation, notably DCL (*r* = –0.782), LIN (*r *= –0.972), STR (*r* = –0.791), and WOB (*r* = –0.687), implying their importance in maintaining DNA integrity. Although VCL and VSL also showed negative correlations with DNA fragmentation (*r* = – 0.786 and – 0.829), emphasis on stronger associations, such as LIN and STR, highlights their critical impact on sperm quality. Additionally, ALH exhibited negative correlations with viability (*r* = – 0.61), membrane integrity (*r* = – 0.56), acrosome integrity (*r *= – 0.92), and DNA fragmentation; however, these were not statistically significant (*p* > 0.05). These findings underscore the pivotal role of specific kinematic parameters, particularly DCL, LIN, STR, and WOB, in determining sperm quality and function in Murrah buffalo bulls.

## Discussion

The present study evaluated the sperm quality and kinematic parameters of Murrah buffalo bulls, with a particular focus on the potential improvements in predicting fertility by integrating traditional semen quality assessments with sperm kinematic analysis. The results reveal substantial insights into the reproductive potential of Murrah buffalo, highlighting both the strengths and limitations of conventional BSE and the advantages of incorporating dynamic sperm movement characteristics.

The results of this research reveal notable differences in sperm quality and kinematic traits among individual bulls, specifically in parameters such as motility, viability, MMP, membrane integrity, and select sperm kinematic measures such as VAP. These findings align with previous reports indicating substantial individual variability in semen quality even within the same breed [24]. This inherent variation carries significant consequences for breeding programs, emphasizing the need for consistent semen quality and reproductive efficiency to maximize fertility success.

A prominent result of this study was the considerable variation in sperm viability and membrane integrity among the bulls examined. These outcomes corroborate the observations by Kumar et al. [12], who reported that bulls exhibiting higher sperm viability and membrane integrity generally achieve superior reproductive outcomes. Sperm viability serves as a vital measure of the sperm’s capacity to survive and function within the female reproductive tract, while membrane integrity indicates the sperm’s fertilization potential [25]. Consequently, the detected disparities in these parameters among the bulls may offer important insights into their fertility capabilities and underscore the necessity for more precise selection strategies within breeding programs [26].

The study also assessed MMP, which is another key indicator of sperm health. The MMP is vital for the energy production required for sperm motility and successful fertilization [27]. The high MMP observed in M004 aligns with previous studies that link higher MMP with better sperm motility and overall fertility potential [28]. The finding suggests that MMP could serve as an important marker for identifying bulls with superior fertility potential, reinforcing the value of incorporating MMP assessments into BSE. Despite having the lowest MMP, bull M003 exhibited the highest motility among the bulls. This discrepancy suggests that factors beyond MMP, such as enhanced ATP utilization efficiency, structural advantages in spermatozoa, or genetic differences, may contribute to sperm motility. These findings highlight the complexity of factors influencing sperm function and emphasize the need for a multifaceted approach to evaluating fertility potential. This reinforces the value of incorporating MMP assessments into breeding soundness evaluations, alongside other key parameters.

In terms of sperm kinematics, the results revealed that while most kinematic parameters did not differ significantly between bulls, VAP exhibited significant variation. These findings support previous research suggesting that VAP is closely correlated with sperm motility and fertilization potential, as it reflects the speed and directionality of sperm movement [13, 29]. This is particularly important, as sperm with higher VAP are more likely to reach and penetrate the egg, a crucial factor in successful fertilization. The significant differences observed in VAP between the bulls in this study may, therefore, offer a more precise measure of sperm fertility potential compared to traditional motility assessments.

The correlation analysis between sperm quality and kinematic parameters revealed several significant relationships that underscore the interconnectedness of sperm health and movement characteristics. Sperm kinematic parameters such as VCL, VAP, and ALH were strongly correlated, reflecting the interdependence of sperm speed and movement precision [3]. The strong positive correlation between VCL and VAP observed in this study is consistent with the findings of Seyoum and Lemma [11], who also noted that sperm with higher VCL tend to have higher VAP and thus greater potential for successful fertilization.

In contrast, some correlations were weak and not statistically significant, such as those between sperm concentration and other quality parameters. Amplitude of lateral head movement (ALH) showed a significant negative association with acrosome integrity, indicating potential damage to the acrosome structure due to extreme movement. Spermatozoa abnormality was also positively correlated with protein deficiency, indicating that abnormal sperm tend to have structural and metabolic weaknesses that reduce their functional quality. Greater lateral movement may indicate inefficient movement or unfocused energy, which is often associated with structural damage to the sperm, such as head or tail abnormalities [24]. This result underscores the importance of considering not just sperm concentration but also dynamic characteristics such as motility, viability, and kinematics in predicting fertility. While sperm concentration remains a critical factor in semen quality assessment, it alone may not be sufficient to reliably predict reproductive success, particularly in cases where sperm motility and other dynamic factors are less optimal [30].

A key finding from the correlation analysis was the strong positive relationship between STR and motility, as well as LIN and motility, as shown in Figure 2C. However, the relationships between BCF and other sperm quality metrics, including motility and membrane integrity, were weak or non-significant (*r* < 0.50). These findings emphasize the importance of STR and LIN as indicators of directed sperm movement and their potential role in enhancing sperm quality. These traits influence the sperm's ability to move effectively and maintain a direct trajectory toward the egg, enhancing the likelihood of successful fertilization. This aligns with Fernandez et al. [7], who reported that sperm with more linear movement patterns (higher STR) have a better chance of successfully navigating the female reproductive tract. In this regard, incorporating kinematic parameters such as STR and LIN into breeding evaluations could provide more accurate predictions of a sperm’s fertilization potential [31].

Despite the promising insights gained from this study, several limitations must be acknowledged. First, while the study provided valuable data on sperm quality and kinematic parameters, it is important to recognize that sperm motility and other kinematic measurements alone may not fully capture all the complexities of sperm behavior during fertilization. As highlighted by Khanal et al. [5], the fertilization process involves numerous additional factors, including sperm–egg interactions, which cannot be assessed through semen analysis alone. Future research could explore the integration of advanced molecular and biochemical assessments to complement sperm quality and kinematic evaluations, providing a more comprehensive picture of sperm fertility potential.

Furthermore, while this study focused on frozen semen, it is important to consider that the freezing and thawing process can affect sperm quality, potentially masking differences in fertility potential that would be evident in fresh semen. Studies examining the effects of cryopreservation on sperm kinematics and quality, Gholami et al. [19] have shown that the freezing process can negatively impact certain parameters, such as membrane integrity and motility. Therefore, the results of this study may not fully reflect the fertility potential of fresh semen from these bulls.

The small sample size (4 bulls) may not fully represent the broader Murrah buffalo population, as this species is a relatively new focus of exploration in Indonesia, with limited availability for research. Additionally, the use of frozen semen, while ensuring uniform laboratory conditions, may not accurately reflect the fertility potential of fresh semen, warranting future studies to compare both. The study also did not evaluate critical factors such as oxidative stress or sperm–egg interaction, which are essential for understanding fertility outcomes. Furthermore, the lack of field-based fertility data, such as pregnancy rates following AI, limits the validation of laboratory findings. Future research should address these gaps by incorporating larger sample sizes, assessing a broader range of sperm quality parameters, and correlating laboratory results with field-based outcomes to strengthen the applicability of the findings.

Despite these limitations, the study’s findings suggest that incorporating sperm kinematics into semen evaluations could significantly improve breeding efficiency and fertility predictions in livestock breeding programs. This approach offers a promising foundation for further studies aimed at enhancing reproductive management and productivity in Murrah buffalo and other livestock species. The findings of this study highlight that integrating conventional semen quality assessments with detailed kinematic analyses through CASA provides a more reliable and comprehensive evaluation of sperm functionality, enabling accurate fertility predictions in Murrah buffalo bulls. CASA significantly enhances the precision of breeding soundness evaluations by providing real-time insights into sperm movement parameters, enabling refined selection criteria for sires with superior fertility potential. These advancements not only optimize genetic selection strategies but also improve breeding program efficiency, leading to increased fertility rates, better herd management, and higher productivity in Murrah buffalo, particularly in milk and meat production. Ultimately, these findings have the potential to enhance milk production and meat yield in Murrah buffaloes while also offering a model for improving reproductive management in livestock species through both natural breeding and AI programs.

## Conclusion

This study demonstrated that integrating conventional semen quality evaluations with detailed sperm kinematic analysis enhances the accuracy of fertility assessment in Murrah buffalo bulls. Crucial kinematic variables, including VAP, VCL, and STR, showed significant correlations with sperm quality, underscoring their relevance in reproductive evaluations. Bulls exhibiting elevated VAP and VCL values also presented superior sperm motility and membrane integrity, factors critical to successful fertilization. These findings emphasize the benefit of incorporating CASA into routine breeding evaluations to achieve more precise measurements of semen parameters, which serve as essential surrogate markers for fertility potential. Implementing this integrated approach could significantly improve the effectiveness and outcomes of buffalo breeding programs.

## List of abbreviations

AIC, Artificial Insemination Center; ANOVA, analysis of variance; CASA, Computer-Assisted Sperm Analysis; CMA3, chromomycin A3; DNA, deoxyribonucleic acid; FITC-PSA, fluorescein-conjugated lectin Pisum sativum agglutinin; HOS, hypo-osmotic swelling; LIN, linearity; MMP, mitochondrial membrane potential; PM, progressive motility; STR, straightness; TM, total motility; VAP, average path velocity; VCL, curvilinear velocity; VSL, straight-line velocity.
